# DNA alkylation and interstrand cross-linking by treosulfan

**DOI:** 10.1038/sj.bjc.6690043

**Published:** 1999-01

**Authors:** J A Hartley, C C O'Hare, J Baumgart

**Affiliations:** CRC Drug–DNA Interactions Research Group, Department of Oncology, UCL Medical School, 91 Riding House Street, London, W1P 8BT, UK; Medac, Fehlandstrasse 3, Hamburg, D-20354, Germany

**Keywords:** treosulfan, DNA cross-linking, DNA alkylation

## Abstract

The anti-tumour drug treosulfan (L-threitol 1,4-bismethanesulphonate, Ovastat) is a prodrug for epoxy compounds by converting non-enzymatically to L-diepoxybutane via the corresponding monoepoxide under physiological conditions. The present study supports the hypothesis that this conversion of treosulfan is required for cytotoxicity in vitro. DNA alkylation and interstrand cross-linking of plasmid DNA is observed after treosulfan treatment, but this is again produced via the epoxide species. Alkylation occurs at guanine bases with a sequence selectivity similar to other alkylating agents such as the nitrogen mustards. In treosulfan-treated K562 cells, cross-links form slowly, reaching a peak at approximately 24 h. Incubation of K562 cells with preformed epoxides shows faster and more efficient DNA cross-linking. © 1999 Cancer Research Campaign


British Journal of Cancer (1999) 79(2), 264–266
© 1999 Cancer Research Campaign

Short communication

### DNA alkylation and interstrand cross-linking by treosulfan

**JA Hartley[1], CC O'Hare[1] and J Baumgart[2]**

[1]CRC Drug–DNA Interactions Research Group, Department of Oncology, UCL Medical School, 91 Riding House Street, London W1P 8BT, UK; [2]Medac, Fehlandstrasse 3, D-20354 Hamburg, Germany

**Summary** The anti-tumour drug treosulfan (L-threitol 1,4-bismethanesulphonate, Ovastat) is a prodrug for epoxy compounds by converting non-enzymatically to L-diepoxybutane via the corresponding monoepoxide under physiological conditions. The present study supports the hypothesis that this conversion of treosulfan is required for cytotoxicity in vitro. DNA alkylation and interstrand cross-linking of plasmid DNA is observed after treosulfan treatment, but this is again produced via the epoxide species. Alkylation occurs at guanine bases with a sequence selectivity similar to other alkylating agents such as the nitrogen mustards. In treosulfan-treated K562 cells, cross-links form slowly, reaching a peak at approximately 24 h. Incubation of K562 cells with preformed epoxides shows faster and more efficient DNA cross-linking.

**Keywords:** treosulfan; DNA cross-linking; DNA alkylation

### INTRODUCTION

Treosulfan (L-threitol 1,4-bismethanesulphonate, Ovastat; Figure 1), synthesized more than 30 years ago (Feit, 1961, 1964), possesses a broad spectrum of anti-tumour activity (White, 1962). The clinical experience with the drug is, however, limited largely to oral and intravenous treatment of patients with advanced ovarian cancer (Lundrall, 1976; Fennelly, 1977; Breitbach et al, 1994), although toxicological evaluation and clinical experience revealed the lack of significant non-haematological toxicity, which suggests treosulfan as a promising candidate for high-dose chemotherapy with autologous stem cell reinfusion.

Although treosulfan shows some structural similarity to the alkylating agent busulphan, its mechanism of alkylation is distinct. Unlike busulphan, which alkylates as a primary methane-sulphonate, treosulfan is a prodrug for epoxy compounds and converts non-enzymatically to L-diepoxybutane via the corresponding monoepoxide under physiological conditions (Feit et al, 1970; Figure 1). The cytotoxicity of treosulfan is assumed to be due to alkylation of DNA and the formation of DNA interstrand cross-links, although this has not been proven. In the present study, the alkylation and cross-linking of DNA is confirmed both in cell-free systems and in intact cells. In addition, the cytotoxicity and cross-linking is confirmed to be due to the conversion of treosulfan to the epoxide species.

### MATERIALS AND METHODS

### Cell culture and cytotoxicity studies

The human chronic myelogenous leukaemic cell line K562 as a suspension culture was grown at 37°C in an atmosphere of 95% air/5% carbon dioxide in RPMI-1640 containing 10% heat-inactivated fetal bovine serum and 2 mM glutamine. Cytotoxicity was assessed in K562 cells using the MTT assay (Carmichael et al, 1987). Cells were cultured in 96-well plates and grown for 4 days before staining with MTT.

### Drug treatment

Treosulfan was obtained as the crystalline preparation for clinical use (Ovastat) from Medac, Hamburg, Germany. Stock solutions (20 mM) of treosulfan were prepared immediately before use in either water or sterile medium. Where appropriate, conversion of treosulfan to the corresponding epoxides was achieved in solution by the addition of sodium hydroxide (Figure 1). This transformation was accomplished by addition of 1.33 mol sodium hydroxide per mol of treosulfan, resulting in a mixture of the corresponding monoepoxide and L-diepoxybutane in approximately equal

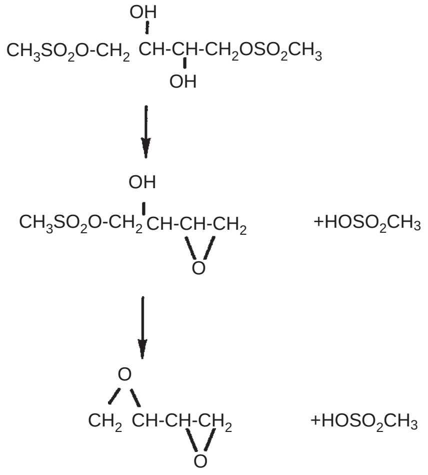

**Figure 1** Structure of treosulfan and its spontaneous pH-dependent activation into the monoepoxide (2S,3S)-1,2-epoxy-3,4 butanediol 4-methanesulphonate and the diepoxide, L-diepoxybutane

Received 20 August 1997
Revised 1 May 1998
Accepted 1 May 1998

Correspondence to: JA Hartley amounts (Feit et al, 1970). The alkaline transformation of aqueous solutions of treosulfan was confirmed by high-performance liquid chromatographic (HPLC) analysis (PW Feit, personal communication).

### DNA interstrand cross-linking in plasmid DNA

*Bam*HI linearized and dephosphorylated pBR322 DNA was 5′-end labelled with T4 polynucleotide kinase and [$\gamma$-$^{32}$P]ATP (5000 Ci mmol$^{-1}$, Amersham), and ~10 ng of labelled DNA was used for each experimental point. Reactions with drug were performed in 25 mM triethanolamine, 1 mM EDTA (pH 7.2) at 37°C and terminated by the addition of an equal volume of stop solution (0.6 M sodium acetate, 20 mM EDTA, 100 μg ml$^{-1}$ tRNA). The samples were precipitated, denatured and electrophoresed exactly as described previously (Hartley et al, 1991).

### *Taq* polymerase stop assay

The procedure was an application of the method of Ponti et al (1991*a*). Before drug/DNA incubation, plasmid pBR322 DNA was linearized with *Hin*dIII to provide a stop for the *Taq* downstream of the primer. The oligodeoxynucleotide primers were 5′-end labelled before amplification using T4 polynucleotide kinase and [$\gamma$-$^{32}$P]ATP (5000 Ci mmol$^{-1}$, Amersham). The labelled primers were purified by elution through BioRad BioSpin columns. The synthetic primer used for amplification of a 330-bp region was 5′-GTCGCCATGATCGGCGTAGTC. Linear amplification of DNA was carried out in a total volume of 100 μl containing 0.5 μg template DNA, 5 pmol labelled primer, 125 μM each dNTP, 1 U *Taq* polymerase, 20 mM diammonium sulphate, 75 mM tris-HCl, pH 9, 0.01% Tween, 2.5 mM magnesium chloride and 0.05% gelatin. After an initial denaturation at 94°C for 1 min, the cycling conditions were as follows: 94°C for 1 min, 60°C for 1 min, and 72°C for 1 min, for a total of 30 cycles. Samples were precipitated and electrophoresed as described previously (Ponti et al, 1991*a*).

### Alkaline elution analysis

Analysis of DNA interstrand cross-link formation in K562 cells was carried out using alkaline elution as described by Kohn et al (1981).

### RESULTS

After a 1-h exposure of human leukaemic K562 cells to treosulfan, there is little effect on cell survival at doses up to 1 mM. In contrast, treatment for 1 h with treosulfan that has been pre-incubated with sodium hydroxide to convert it to the corresponding monoepoxide and L-diepoxybutane (Figure 1) showed significant cell killing, with an $IC_{50}$ (dose of drug producing 50% loss in cell survival) of 115 μM. If, however, treosulfan is administered continuously to the cells during the cell survival assay, cell killing is observed with an $IC_{50}$ of 75 μM. Under these conditions, the epoxide mixture was again more cytotoxic with an $IC_{50}$ value of 20 μM, but the difference between the parent drug and the pretransformed epoxides was much less.

The ability of treosulfan to produce DNA interstrand cross-links was tested using a sensitive agarose gel-based plasmid DNA assay (Figure 2). Treosulfan was able to produce cross-links after a 4-h exposure, but 10 mM drug was required to produce 8.9% crosslinking (Figure 2B). With a 24-h exposure to treosulfan (Figure 2D), cross-linking at 10 mM had increased to 62.2%, with 11.6% cross-linking at 1 mM. In contrast, significant cross-linking was observed with the preformed epoxides at 4 h (Figure 2A), with 29.7% cross-linking at 10 mM increasing to 90.7% with a 24-h exposure (Figure 2C).

The sequence specificity of covalent binding to DNA by treosulfan was examined using a *Taq* polymerase stop assay (Figure 3). After a 4-h exposure to treosulfan, significant blocks to the polymerase were only observed at 10 mM (lane E). With the preformed epoxides, a similar level of blockage was observed with 1 mM (lane B), and at 10 mM (lane C) extensive evidence of covalent binding was observed. Examination of the sites of blockage revealed alkylation at guanine bases with a preference for runs of contiguous guanines, as has been observed previously with alkylating agents such as nitrogen mustards. The pattern of damage was different to cisplatin (lane F), which produced blocks primarily at GG and AG sites (Ponti et al, 1991*a*). Alkylation by the epoxides was confirmed as guanine-N7 alkylation using a piperidine cleavage-based sequencing assay (data not shown).

DNA cross-linking in K562 cells was investigated using alkaline elution. After a 1-h exposure of cells to treosulfan followed by a 4-h incubation in drug-free medium, cross-links were just detectable at 10 μM treosulfan with increased levels at 100 μM (data not shown). A higher level of cross-links was observed under identical conditions when the cells were treated with 10 μM of the preformed epoxides, which increased at 100 μM. In unirradiated samples, no evidence of drug-induced single-strand breakage was observed with either agent (data not shown). When cells were treated with treosulfan continuously, cross-links formed slowly and were still increasing at 15 h, consistent with the slow non-enzymatic conversion of treosulfan to the diepoxide.

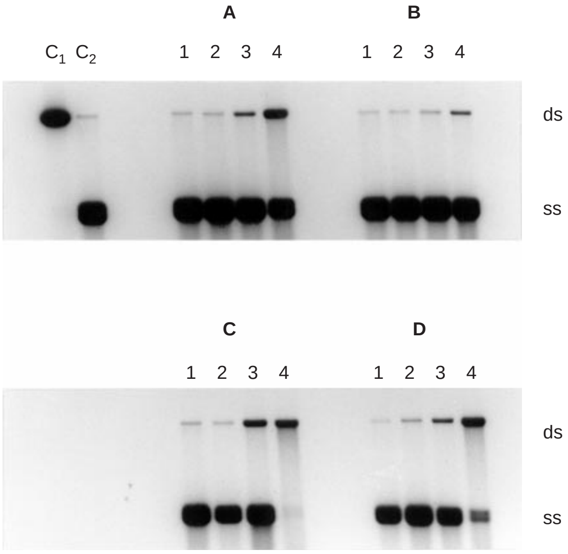

**Figure 2** DNA interstrand cross-linking by treosulfan in plasmid DNA. Drug treatment was either for 4 h (**A**, **B**) or 24 h (**C**, **D**). Treosulfan was either administered directly (**B**, **D**) or after conversion to the corresponding monoepoxide and L-diepoxybutane (**A**, **C**). The dose of drug was 0.01 mM (lane 1), 0.1 mM (lane 2), 1 mM (lane 3), or 10 mM (lane 4). $C_1$ is control, non-denatured DNA and $C_2$ is control, denatured DNA. Samples in lanes 1–4 were denatured before gel loading; ds and ss are double-stranded and single-stranded DNA respectively

© Cancer Research Campaign 1999

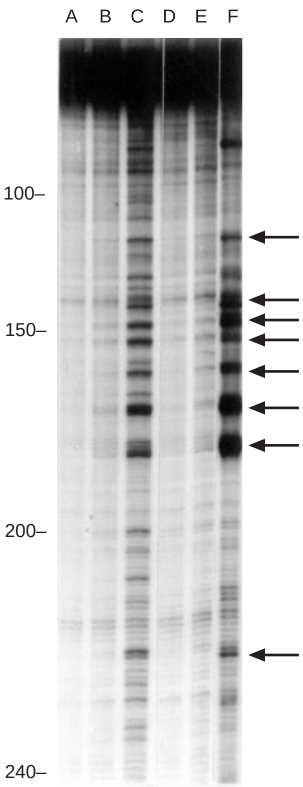

**Figure 3** Sequence specificity of alkylation of plasmid DNA by treosulfan. Lane A, control, undamaged plasmid; lane B, epoxide mixture, 1 mM; lane C, epoxide mixture, 10 mM; lane D, treosulfan, 1 mM; lane E, treosulfan, 10 mM; lane F, cisplatin, 50 μM. Drug reactions were for 4 h at 37°C. Numbers refer to the base sequence position in pBR322 DNA and arrows indicate the position of runs of two or more contiguous guanine bases

### DISCUSSION

Alkylation and interstrand cross-linking of DNA by treosulfan has been demonstrated in cell-free systems and in intact cells. Conversion of treosulfan to epoxide species (Figure 1) is required for alkylation and cross-linking to occur and resultant cytotoxicity. Although structurally similar to busulphan, its mechanism of alkylation is, therefore, distinct. However, the ultimate sites of alkylation on DNA are identical to those previously described for busulphan (Ponti et al, 1991*b*). The initial alkylation event produced by treosulfan on DNA is primarily at the guanine N-7 position with the preference for contiguous runs of guanines. This has previously been shown for other bifunctional agents such as nitrogen mustards (Mattes et al, 1986; Hartley, 1993) and busulphan (Ponti et al, 1991*b*). The four carbon cross-links produced by treosulfan should span the same distance on DNA as busulphan (6.0 Å), which is unlikely to be between two guanine N-7 positions because the shortest distance between N7 atoms on opposite strands is approximately 8.0 Å in B-form DNA. The rate of cross-link formation by busulphan is slow and still increasing at 24 h (Ponti et al, 1991*b*). With treosulfan, cross-link formation in naked DNA and cells is also slow, but this reflects the slow conversion of treosulfan to the active epoxide species.

Alkylation of DNA requires conversion of treosulfan to the monoepoxide and cross-linking can only occur via a diepoxide species. In vitro, cross-linking is clearly at sub-millimolar concentrations of drug. In patients, peak plasma levels of treosulfan reach ~2 mM after conventional doses of 8–10 g $m^{-2}$ over a 0.5-h infusion (Hilger et al, 1998). Levels of ~4 mM are achieved after high-dose therapy with stem cell support at a dose of 32.5 g $m^{-2}$ given as a 2-h infusion (Scheulen et al, 1997). Until now, there are no published reports concerning the levels of the corresponding epoxide species in the plasma of patients undergoing treosulfan therapy. Possibly, the lipophilicity of the epoxides is resulting in a rapid disappearance from the first compartment. A bolus injection of L-diepoxybutane to dogs led to the rapid disappearance of the compound within minutes even though the chemical stability of L-diepoxybutane in plasma is high ($T_{1/2}$ = 7 h ex vivo) (PW Feit, personal communication).

Since its initial synthesis over 30 years ago, the mechanism of action of treosulfan has been assumed to be as a bifunctional alkylating agent after conversion non-enzymatically to the epoxide species. The present study confirms this mechanism.

### ACKNOWLEDGEMENTS

The authors acknowledge Dr PW Feit for helpful discussions. This study was funded in part by the Cancer Research Campaign.

### REFERENCES

Breitbach GP, Villena C, Schwickerath J, Kunz T, Hurst U, Schmidt-Rhode P, Friedrich EJ, Artmeyer E, Schröder M, Eberl M, Franck J, Meinen K, Schiller S, Wernicke K, Sasse G, Schmid H and Bastert G (1994) Treosulfan bei fortgeschrittenem Ovarialcarcinom: eine Phase III-studie. *Arch Gynecol Obstet* **225** (suppl. 1): 80

Carmichael J, De Graff WG, Gazdar AF, Minna JD and Mitchell JB (1987) Evaluation of a tetrazolium based semi-automated colorimetric assay. I. Assessment of chemosensitivity testing. *Cancer Res* **47**: 936–940

Feit PW (1961) Stereoisomere 1,4-di-*O*-methanesulfonyl-butan-2,3,4,4-tetrole. *Tetrahedron Lett* **20**: 716–717

Feit PW (1964) 1,4-Bismethanesulfonates of the steroisomeric butane tetrads and related compounds. *J Med Chem* **7**: 14–17

Feit PW, Rastrup-Andersen N and Matagne R (1970) Studies on epoxide formation from (2S,3S)-threitol 1,4-bismethanesulfonate. The preparation and biological activity of (2S,3S)-1,2-epoxy-3-4-butanediol 4-methanesulfonate. *J Med Chem* **13**: 1173–1175

Fennelly JJ (1977) Treosulfan in the management of ovarian carcinoma. *Br J Obstet Gynecol* **84**: 300–303

Hartley JA (1993) Selectivity in alkylating agent DNA interactions. In *Molecular Aspects of Anticancer Drug DNA interactions*. Neidle S and Waring MJ (eds.), pp. 1–31. Macmillan Press: Basingstoke

Hartley JA, Berardini MD and Souhami RL (1991) An agarose gel method for the determination of DNA interstrand crosslinking applicable to the measurement of the rate of total and 'second-arm' crosslink reactions. *Anal Biochem* **193**: 131–134

Hilger RA, Harstrick A, Eberhardt W, Oberhoff C, Skorzec M, Baumgart J, Seebers S and Scheulen ME (1998) Clinical pharmacokinetics of intravenous treosulfan in patients with advanced solid tumors. *Cancer Chemother Pharmacol* **42**: 99–104

Kohn KW, Ewig RAG, Erickson LC and Zwelling LA (1981) Measurement of strand breaks and crosslinks by alkaline elution. In *DNA Repair*, vol 1B. Friedberg EC and Hanawalt PC (eds.), pp. 379–403. Dekker: New York

Mattes WB, Hartley JA and Kohn KW (1986) DNA sequence selectivity of guanine N7 alkylation by nitrogen mustards. *Nucl Acids Res* **14**: 2971–2987

Ponti M, Forrow SM, Souhami RL, D'Incalci M and Hartley JA (1991*a*) Measurement of the sequence specificity of covalent DNA modification by antineoplastic agents using *Taq* DNA polymerase. *Nucl Acids Res* **19**: 2929–2933

Ponti M, Souhami RL, Fox BW and Hartley JA (1991*b*) DNA interstrand crosslinking and sequence selectivity of dimethanesulphonates. *Br J Cancer* **63**: 743–747

Scheulen ME, Hilger RA, Harstrick A, Oberhoff C, Freund M, Casper J, Skorzec M, Baumgart J and Seebers (1997) Clinical pharmacology of dose escalation of intravenous treosulfan with autologous stem cell support in patients with advanced solid tumors. *Eur J Clin Pharmacol* **52** (suppl.): A107

White FR (1962) L-Threitol dimethanesulfonate. *Cancer Chemother Rep* **24**: 95–97

*© Cancer Research Campaign 1999*

